# Evaluation of adverse effects with COVID-19 vaccination in Pakistan

**DOI:** 10.12669/pjms.37.7.4522

**Published:** 2021

**Authors:** Sana Abbas, Beenish Abbas, Sidra Amir, Mehreen Wajahat

**Affiliations:** 1 Dr. Sana Abbas, MCPS. Gwadar Development Authority Hospital, Gwadar, Pakistan; 2 Dr. Beenish Abbas, FCPS. Foundation University, Islamabad, Pakistan; 3 Dr. Sidra Amir, MBBS. Foundation University, Islamabad, Pakistan; 4 Dr. Mehreen Wajahat, MPhil. Avicenna Medical and Dental College, Lahore, Pakistan

**Keywords:** Covid-19, Vaccine, Adverse effects

## Abstract

**Objectives:**

Vaccinations work with different mechanisms to offer protection against disease; however, process of immunity building can cause symptoms. Therefore, this study aimed to determine the immediate side effects of COVID–19 vaccination in the Pakistani Population.

**Methods:**

This cross-sectional analytical study was conducted at Foundation University College of Dentistry, Islamabad from February to April 2021. 0.5 mL per dose of the Covid-19 vaccine was administered to the candidates. These 205 candidates receiving vaccination were then interviewed investigating the adverse effects of the vaccine. Post-vaccination side effects were compared among categorical groups using the Chi-Square test, whereas post-vaccination side effects were compared with age using independent samples T-test. A p-value of ≤0.05 was statistically significant.

**Results:**

Among post-vaccination side effects, fever was reported by 69 participants, while 56 of 205 reported soreness, redness, and swelling at the injection site. It was reported by 42/205 participants to have felt chills and rigor, whereas gastrointestinal disturbance and flu-like symptoms were reported in 55/205 and 28/205 participants, respectively. Younger participants were more likely to develop gastrointestinal disturbance and flu-like symptoms following vaccination as compared to older participants.

**Conclusion:**

Malaise, headache, and fever were observed to be the most common side effects of the vaccine, moreover there was a linear relationship between manifestations of adverse effects and history of comorbidities.

## INTRODUCTION

COVID-19 has caused not only enormous deaths but also the recession to economies of nations.^1^ The risk of outbreaks and disruption to economic and social life will probably remain until effective vaccines are administered at a mass level to the global population.^2^ Data released from the World Health Organization on November 12, 2020, there were 212 vaccines under testing, inactivated or attenuated vaccines, traditional type of vaccine, genetically engineered recombinant adenovirus vector vaccine, ribonucleic acid (RNA) vaccines, recombinant viral vector vaccine, and deoxyribonucleic acid (DNA) vaccines.^3^

Commendable efforts since the identification of the COVID-19 genome led to the development of over 300 vaccine projects. According to recent studies, 40 are now in the clinical evaluation phase, more than 10 of these vaccines under question are in phase III clinical trials, three of these have successfully passed phase III trial evaluations.^4^ Due to the novelty and diversity of technological platforms exploited, vaccine efficacy and safety remain to be elucidated. Further research is needed to evaluate the effectiveness of vaccines in imparting immunological defenses by training innate immunity to Corona virus and pathogen agonist protection.^5^

A published study from vaccination results from Israel’s vaccination rollouts from December to February demonstrated that two doses of Pfizer-BioNTech vaccine reduced hospital admissions by 87%, symptomatic cases by 94%, hospital admissions by 87%.This study also revealed vaccine effectiveness against the B.1.1.7 variant; however, there was a sparsity of data on the vaccine’s effectiveness against B.1.351 variant.^6^ Pfizer and Oxford-AstraZeneca are reportedly updating their vaccine against newly discovered variants in Finland and Germany, while Moderna is still waiting for approval from regulators to carry out trials of modified version for effective targeting of B.1.351 variant.^7^

King’s College London found that local and few “after-effects” are experienced after their 1^st^ and 2^nd^ dose.^4^,^8^ Rationale behind this study was to evaluate the frequency of adverse effects of Sino-pharm Vaccination administered to the Pakistani Population.

## METHODS

This Cross-sectional analytical study was conducted at Foundation University College of Dentistry (FUCD) from February to April 2021. Ethical approval was taken from the Ethical Review Committee, of the institute (Ref: FF/FUMC/215-110/Phy/21, Dated: March 11, 2021). Sample size (205) was calculated using the World Health Organization (WHO) sample size calculator and keeping a level of significance 95% confidence interval, 5% error, and anticipated prevalence of fever 15.8 % was determined using a study conducted by the Centre for disease control and prevention.^9^ Eighten to fifty year old subjects undergoing Sino-pharm coronavirus vaccination, provided by the Chinese Government to Pakistan, with ASA I & II status, hemodynamically stable with optimized comorbid were recruited in the study. Pregnant patients, those with decompensated diabetic and hypertensive profile, known asthmatics, immunocompromised patients, older than 65 years of age, unwilling for vaccination, had a Covid–19 disease history in last 03 months, SARS-CoV-2 infection specific IgG or IgM positive in serum; positive PCR test for SARS-CoV-2 infection from a pharyngeal or anal swab sample; the axillary temperature of more than 37ºC, allergic to drug contents, having a specific contraindication to vaccinations, and BMI > 35 Kg/m^2^ were excluded from this study.

As per study protocol, all the patients were briefed about the procedure and informed written consent was taken. Before vaccination, a detailed general physical examination was carried out in all patients with necessary laboratory evaluation parameters, including negative PCR test results, to adhere to our inclusion and exclusion criteria. In the vaccination room equipped with emergency resuscitation facilities, reporting the candidate’s name, age, and contact details were endorsed in the record before vaccination. In addition, vital signs and recent history of respiratory tract infections were recorded. 0.5 mL per dose of coronavirus inactivated Vaccine in ready-to-use syringes was administered intramuscularly in the deltoid muscle. After vaccination, each candidate was kept under observation for 30 minutes. Candidates were then provided with adverse effects paper-based proforma devised by relevant studies on this subject. In addition to demographic profile questionnaire incorporated history of coronavirus disease, comorbids profile, and willingness for vaccination inoculation. Adverse effects inquired in the questionnaire are enumerated in results section and surveillance of adverse effects was continued for seven days after administration of vaccination.

Data were statistically analyzed using IBM SPSS (version 23.0). The descriptive statistics of continuous variables were presented as mean and standard deviation, while categorical data, frequencies and percentages were used. Post-vaccination side effects were compared among categorical groups, including gender, comorbidities, and history of COVID-19 infection, using the Chi-Square test, whereas post-vaccination side effects were compared with age using independent samples T-test. A p-value of ≤0.05 was statistically significant.

**Fig.1:**
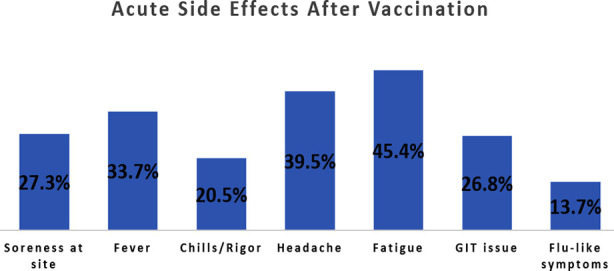
Distribution of commonly reported post-vaccination acute side effects.

## RESULTS

There were 205 participants included in this study, all of whom were vaccinated for COVID-19 viral infection. The mean age of study participants was 32.96±7.7 years, with an age range of 23 – 55 years. There were 88 (42.9%) males and 117 (57.1%) females. There were 23 (11.2%) participants with a history of diabetes, 25 (12.2%) had hypertension, while two (1.0%) had a history of asthma. Around 19.5% (40/205) of the participants were ever tested positive for COVID-19 infection in the recent past, whereas 60 (29.3%) were currently experiencing symptoms of COVID-19 including sore throat/flu-like symptoms. The baseline characteristics of study participants are summarized in Table-I.

**Table I:** Baseline characteristics of study participants (n=205).

*Characteristics*		*Frequency (n)*	*Percentage (%)*
Age in years (mean ± SD)		32.96±7.7	
Age range		23 – 55	
Gender	Male	66	42.9%
	Female	117	57.1%
Comorbidities	Diabetes	23	11.2%
	Hypertension	25	12.2%
	Asthma	2	1.0%
Tested positive for COVID-19	Yes	40	19.5%
	No	165	80.5%
Overall experienced symptoms	Yes	60	29.3%
	No	145	70.7%

In terms of post-vaccination symptoms, the distribution of acute side effects is presented in Fig.1.

The reported post-vaccination side effect was fatigue/malaise reported by 45.4% (93/205) of the participants, followed by 39.5% (81/205) headache/migraine. Fever was reported by 33.7% (69/205) of the participants post-vaccination, while 27.3% (56/205) participants reported soreness, redness, and swelling at the injection site. In addition, it was reported by 20.5% (42/205) participants to have felt chills and rigor, whereas gastrointestinal disturbance and flu-like symptoms were reported in 26.8% (55/205) and 13.7% (28/205) participants, respectively.

Comparisons of post-vaccination acute side effects with demographic characteristics including age, gender, comorbidities, history of COVID-19 infection, and symptoms at the time of vaccination are illustrated in Table-II and Table-III. Age was significantly associated with post-vaccination side effects, including gastrointestinal disturbance and flu-like symptoms. In addition, it was found that younger participants were more likely to develop gastrointestinal disturbance following vaccination as compared to older participants (31.3±7.4 vs 33.8±7.8, p=0.040). Similarly, flu-like symptoms were more likely to be reported in younger participants than older ones (36.0±6.4 vs 32.6±7.9, p=0.030).

**Table II:** Comparison of post-vaccination acute side effects with age and gender.

*Post-vaccination side effects*	*Mean Age*	*P*	*Gender*		*P*	*History of COVID infection*		*P*
	
			*Male*	*Female*		*Yes*	*No*	
***Soreness/Redness***								
• Yes (n=56)	34.8±6.3	0.063	16 (28.6%)	40 (71.4%)	0.011*	16 (28.6%)	40 (71.4%)	0.045
• No (n=149)	32.5±8.2		72 (48.3%)	77 (51.7%)		24 (16.1%)	125 (83.9%)	
***Fever***								
• Yes (n=69)	34.5±5.1	0.072	26 (37.7%)	43 (62.3%)	0.280	20 (29.0%)	49 (71.0%)	0.015
• No (n=136)	32.4±8.8		62 (45.6%)	74 (54.4%)		20 (14.7%)	116 (85.3%)	
***Chills/Rigor***								
• Yes (n=42)	33.5±6.1	0.689	14 (33.3%)	28 (66.7%)	0.159	12 (28.6%)	30 (71.4%)	0.097
• No (n=155)	33.0±8.2		74 (45.4%)	89 (54.6%)		28 (17.2%)	135 (82.8%)	
***Headache***								
• Yes (n=81)	33.9±7.3	0.233	28 (34.6%)	53 (65.4%)	0.050*	24 (29.6%)	57 (70.4%)	0.003
• No (n=116)	32.5±8.1		60 (48.4%)	64 (51.6%)		16 (12.9%)	108 (87.1%)	
***Fatigue/Malaise***								
• Yes (n=93)	32.7±7.0	0.522	36 (38.7%)	57 (61.3%)	0.266	26 (28.0%)	67 (72.0%)	0.005
• No (n=104)	33.4±8.5		52 (46.4%)	60 (53.6%)		14 (12.5%)	98 (87.5%)	
***GI disturbance***								
• Yes (n=55)	31.3±7.4	0.040*	30 (54.5%)	58 (38.7%)	0.042*	20 (36.4%)	35 (63.6%)	<0.001
• No (n=142)	33.8±7.8		58 (38.7%)	92 (61.3%)		20 (13.3%)	130 (86.7%)	
***Flu-like symptoms***								
• Yes (n=28)	36.1±6.4	0.030*	12 (42.9%)	16 (57.1%)	0.994	0 (0%)	28 (100%)	0.005
• No (n=169)	32.6±7.9		76 (42.9%)	101 (57%)		40 (22.6%)	137 (77.4%)	

* Significant p-values.

**Table III:** Comparison of post-vaccination acute side effects with comorbidities.

*Post-vaccination side effects*	*Diabetes*		*P-value*	*Hypertension*		*P-value*
	
	*Yes*	*No*		*Yes*	*No*	
***Soreness/Redness***						
• Yes (n=56)	4 (7.1%)	52 (92.9%)	0.257	12 (21.4%)	44 (78.6%)	0.013*
• No (n=149)	19 (12.8%)	130 (87.2%)		13 (8.7%)	136 (91.3%)	
***Fever***						
• Yes (n=69)	5 (7.2%)	64 (92.8%)	0.199	11 (15.9%)	58 (84.1%)	0.243
• No (n=136)	18 (13.2%)	118 (86.8%)		14 (10.3%	122 (89.7%)	
***Chills/Rigor***						
• Yes (n=42)	8 (19.0%)	34 (81.0%)	0.071	6 (14.3%)	19 (11.7%)	0.642
• No (n=155)	15 (9.2%)	148 (90.8%)		19 (11.7%)	144 (88.3%)	
***Headache/Migraine***						
• Yes (n=81)	12 (14.8%)	69 (85.2%)	0.187	24 (29.6%)	57 (70.4%)	<0.001*
• No (n=116)	11 (8.9%)	113 (91.1%)		1 (0.8%)	123 (99.2%)	
***Fatigue/Malaise***						
• Yes (n=93)	6 (6.5%)	87 (93.5%)	0.049*	4 (4.3%)	89 (95.7%)	0.002*
• No (n=104)	17 (15.2%)	95 (84.8%)		21 (18.8%)	91 (81.3%)	
***GI disturbance***						
• Yes (n=55)	6 (10.9%)	49 (89.1%)	0.932	8 (14.5%)	47 (85.5%)	0.533
• No (n=142)	17 (11.3%)	133 (88.7%)		17 (11.3%)	133 (88.7%)	
***Flu-like symptoms***						
• Yes (n=28)	7 (25.0%)	21 (75.0%)	0.013*	1 (3.6%)	27 (96.4%)	0.133
• No (n=169)	16 (9.0%)	161 (91.0%)		24 (13.6%)	153 (86.4%)	

* Significant p-values.

Gender was significantly associated with soreness at the injection site, headache, and gastrointestinal issues. Females were more likely to experience soreness at the site of injection (p=0.011), headache/migraine (p=0.05), whereas males were more likely to experience gastrointestinal disturbance (p=0.042) post-vaccination.

The clinical history of comorbidities was also significantly associated with the development of post-vaccination side effects. For example, diabetic participants were more likely to experience fatigue/malaise (p=0.049) and flu-like symptoms (p=0.013) as compared to non-diabetic’s post-vaccination. Similarly, hypertensive participants more commonly experienced soreness at the site of injection (p=0.013), headache/migraine (p<0.001), and fatigue/malaise (p=0.002) following vaccination.

Participants with previous history of COVID-19 infection were found to be less likely to develop soreness at site of injection (p=0.045), fever (0.015), headache/migraine (p=0.003), fatigue/malaise (p=0.005), gastrointestinal disturbance (p<0.001) and flu-like symptoms (p=0.005) post-vaccination.

## DISCUSSION

To fight the COVID-19 pandemic’s devastating effects on humanity, it is important to administer safe and effective Covid-19 vaccinations and anticipate its side effects in our population. The current study results suggest that side effects are common with vaccines and vary in different vaccines, but few side effects are reported with the Sino-pharm vaccination.

In this study, the Sino-pharm vaccine was well accepted in all age groups (23- 55 years) with no severe side effects. The most common post-vaccination side effect was fatigue/malaise as reported by 45.4% (93/205) of the participants, followed by 39.5% (81/205) headache/migraine. A similar vaccine, Sino-pharm was tested in China, and it did not report any fatigue, headache, feverish and muscle ache but 14.3% localized pain and 2.4% fever were reported.^10^ The variations in the side effects may be due to different contexts and populations. Vaccination trials are being conducted in limited countries, including Europe, the United States, Australia, and China, so further evaluation should be done in other contexts and different age groups. A clinical trial was conducted in the UK with AstraZeneca, and side effects were analyzed;70% of participants reported fatigue, 68% headache, 60% muscle ache, and 51% reported feeling feverish.^11^Another study was conducted in the United States with BioNTech-Pfizer, 83.3% of participants reported post-vaccination fatigue, 100% experienced headache and localized pain, 58.3% muscle ache, and 66.7% feeling feverish.^12^ These findings support few side effects with the Sino-pharm vaccination. No participant developed any symptoms of SARS-CoV-2 infection during the trial. Bhopal et al. found similar results in another study.^13^ Therefore, the inactivated Sino-pharm vaccine in the current research suggests a relatively better safety profile compared with other available vaccines. However, these comparisons should be more carefully inferred as some studies contain a minimal sample size (45)^12^, and some studies reported serious adverse effects.^14^ Few side effects are common with most of the vaccines and they usually mimic coronavirus symptoms. Literature indicates a considerable low incidence of side effects in Sino-pharm and Sinovac vaccines.^10^,^13^

This study has many exclusion criteria so the effects of this vaccine on a wider population requires consideration. Generally, the common side effects of the covid-19 vaccine are mild, like in the current study; however, there is one report of hospitalization after covid-19 vaccination by Johnson and Johnson, which raises queries regarding diagnosing cases of the disease soon after vaccination. The development of transient neutropenia and lymphopenia reported in some trials need consideration.^14^

Data regarding adverse effects of Covid-19 vaccination is limited. According to the media, AstraZeneca trial was stopped twice.^11^ The reported side effects comprise multiple sclerosis and amyotrophic lateral sclerosis, but no further details are known yet.^11^ Brazil stopped the Sinovac trial. No serious side effects have been reported with Sino-pharm in the literature.^13^ There is widespread skepticism in the general population about the Covid-19 vaccine, which is a hurdle in the development of herd immunity through the vaccine,^15^ minimal side effects as reported in our study will help to reduce apprehension among people towards the Covid-19 vaccines. A vaccine that reduces the number of cases should be adopted widely at the population level to reduce the risk of adverse outcome.^16^

Researchers across the globe have invested huge resources towards the development of an effective vaccine, there are incredibly positive reports from phase III clinical trials in terms of efficacy and safety.^17^ Yet the approved vaccines will face challenges that the general population will be skeptical over wider adoption of vaccines due to their novelty. Literature reports suggest that 82% of the population of any given county must be vaccinated to develop herd immunity however, experts acknowledge that there is widespread hesitancy towards vaccine as per initial reports. No serious adverse effects reported by the present study will help reduce vaccine hesitancy. Many countries worldwide like France, Russia, and Poland are reporting a high level of vaccine hesitancy in initial studies.^18^

The findings of this study will help researchers, health specialists, and the public to get the safe Sino-pharm vaccinations without any anxiety as it contains minimum side effects in the Pakistani population.

### Limitations of the study

It includes sample size is very small. Based on such small sample size it is hard to conclude the eventual adverse effect profile of the COVID – 19 vaccinations. Additionally, these study results were only conducted on the patients inoculated with Sinopharm vaccination, therefore, analysis of other available vaccinations cannot be evaluated. With widespread availability of antibody testing facilities, this part of vaccination efficacy could had been incorporated.

## CONCLUSION

Malaise, headache, and fever were observed to be the most common side effects of the vaccine, moreover there was a linear relationship between manifestations of adverse effects and history of comorbidities. To have a clearer picture of the side effects of Covid-19 Vaccines, large sample sized, and multicenter studies are required. Another potential research area is evaluating vaccine efficacy, adverse effects and reporting new Covid 19 cases despite being vaccinated in more vulnerable old age populations.

### Authors Contribution:

**SA:** Conceived, designed and manuscript writing.

**BA, SA & MW:** Data collection, Manuscript drafting and proofreading.

All authors are responsible and accountable for the accuracy or integrity of the work.
